# Pharmacist-led surgical medicines prescription optimization and prediction service improves patient outcomes - a machine learning based study

**DOI:** 10.3389/fphar.2025.1534552

**Published:** 2025-03-14

**Authors:** Xianlin Li, Xiunan Yue, Lan Zhang, Xiaojun Zheng, Nan Shang

**Affiliations:** ^1^ Department of Pharmacy, The First Hospital of Shanxi Medical University, Taiyuan, Shanxi, China; ^2^ School of Pharmacy, Shanxi Medical University, Taiyuan, Shanxi, China; ^3^ School of Public Health, Capital Medical University, Beijing, China

**Keywords:** medication errors, machine learning, pharmacists, patient safety, surgical procedures

## Abstract

**Background:**

Optimizing prescription practices for surgical patients is crucial due to the complexity and sensitivity of their medication regimens. To enhance medication safety and improve patient outcomes by introducing a machine learning (ML)-based warning model integrated into a pharmacist-led Surgical Medicines Prescription Optimization and Prediction (SMPOP) service

**Method:**

A retrospective cohort design with a prospective implementation phase was used in a tertiary hospital. The study was divided into three phases: (1) Data analysis and ML model development (1 April 2019 to 31 March 2022), (2) Establishment of a pharmacist-led management model (1 April 2022 to 31 March 2023), and (3) Outcome evaluation (1 April 2023 to 31 March 2024). Key variables, including gender, age, number of comorbidities, type of surgery, surgery complexity, days from hospitalization to surgery, type of prescription, type of medication, route of administration, and prescriber’s seniority were collected. The data set was divided into training set and test set in the form of 8:2. The effectiveness of the SMPOP service was evaluated based on prescription appropriateness, adverse drug reactions (ADRs), length of hospital stay, total hospitalization costs, and medication expenses.

**Results:**

In Phase 1, 6,983 prescriptions were identified as potential prescription errors (PPEs) for ML model development, with 43.9% of them accepted by prescribers. The Random Forest (RF) model performed the best (AUC = 0.893) and retained high accuracy with 12 features (AUC = 0.886). External validation showed an AUC of 0.786. In Phase 2, SMPOP services were implemented, which effectively promoted effective communication between pharmacists and physicians and ensured the successful implementation of intervention measures. The SMPOP service was fully implemented. In Phase 3, the acceptance rate of pharmacist recommendations rose to 71.3%, while the length of stay, total hospitalization costs, and medication costs significantly decreased (*p* < 0.05), indicating overall improvement compared to Phase 1.

**Conclusion:**

SMPOP service enhances prescription appropriateness, reduces ADRs, shortens stays, and lowers costs, underscoring the need for continuous innovation in healthcare.

## 1 Background

Optimizing prescription practices for surgical patients is critical due to the complexity and sensitivity of their medication regimens. Surgical patients often require multiple medications to manage their conditions before and after surgery, especially elderly patients, who typically need more prescriptions due to comorbidities (Cross et al., 2020). The frequent use of high-alert medications such as anticoagulants (Raccah et al., 2021), opioids (Heneka et al., 2018), anesthesia (Kothari et al., 2010), and sedatives, during surgery increases the risk of prescription errors (Abbasi et al., 2022). Studies report a 9.3% error rate among all prescription in 1,000 perioperative patients in the United States (Langlieb et al., 2023). These errors can lead to adverse drug events (ADEs), prolonged hospital stays, and higher healthcare costs (Durand et al., 2024), highlighting the need to optimize prescriptions to improve outcomes.

Given the high risk of errors, it is crucial to explore strategies to enhance prescription accuracy. Strengthening the clinical role of clinical pharmacists is one such effective strategy (Jia et al., 2021). Previous literature has confirmed that clinical pharmacists play a pivotal role in drug management and reducing ADRs (Johns et al., 2024; Mahomedradja et al., 2023; Delgado-Pérez et al., 2023). Studies have shown that clinical pharmacists have significantly reduced drug errors and adverse drug reactions through systematic drug review services. For instance, research has demonstrated that pharmacist-led prescription review services improve appropriateness, reduce errors, enhance patient outcomes, and minimize resource wastage (Dalton and Byrne, 2017; Jaam et al., 2021; Jošt et al., 2024). Yang, for example, found that a pharmacist-led, system-assisted review model reduced inpatient prescribing errors from 6.94% to 1.96% (Yang et al., 2021). Pharmacists are particularly important in the drug management of surgical patients, especially in complex treatment plans (Xie et al., 2023). Pharmacists can optimize drug selection and administration routes, reduce inappropriate drug use, and thus mitigate the occurrence of drug-related adverse reactions (Nguyen et al., 2023). Other studies have shown that pharmacist-involved drug optimization services significantly reduce the incidence of adverse drug reactions and shorten hospital stays (Gray et al., 2023).

However, while these interventions are beneficial, addressing potential prescription errors (PPEs) is even more critical. Numerous studies have shown that pharmacists can resolve possible conflicts in treatment plans between doctors and pharmacists through effective communication, further improving the safety of drug therapy (Kim et al., 2024; Waszyk-Nowaczyk et al., 2021). Yet over 50% of PPEs identified by pharmacists were not accepted by prescribers in prior studies (Alipour et al., 2018; Albassam et al., 2020). Few studies have developed management models to address unaccepted PPEs, leading to higher risks and potential harm.

Developing a management model for unaccepted PPEs has proven challenging due to factors such as patient conditions, disease complexities, and medication regimens Unresolved PPEs present significant risks to medication safety and efficacy. Prescribers may reject pharmacists’ recommendations due to differing focuses, with prescribers prioritizing outcomes and pharmacists emphasizing drug properties and interactions (Waszyk-Nowaczyk et al., 2021). Heavy workloads and existing management processes contribute to communication discrepancies (Rosen et al., 2018). A study found that 60% of pharmacist interventions were not implemented by prescribers, often due to differences in clinical focus (De Rijdt et al., 2008). The lack of a structured approach exacerbates these gaps in medication safety.

To bridge these challenges, recent advances in machine learning (ML) offer promising solutions. ML can handle large-scale datasets and identify factors influencing healthcare management outcomes. A study demonstrated that models such as Random Forest (RF) and Support Vector Machine (SVM) could enhance predictive accuracy and patient outcomes by identifying critical factors like age, BMI, and blood pressure in diabetic groups (Tan et al., 2023). In recent years, the application of ML in drug management has also gradually increased. Studies have shown that by introducing ML models, pharmacists can more accurately identify potential medication errors and inappropriate drug treatment plans, thereby improving the efficiency and accuracy of drug reviews (Chalasani et al., 2023). For example, by using an RF model, pharmacists can significantly enhance the accuracy and efficiency of drug reviews, consequently reducing drug-related adverse events (Vora et al., 2023; Li et al., 2024). Integrating machine learning models with pharmacists' clinical expertise can help mitigate disagreements between pharmacists and prescribing physicians (Al Meslamani, 2023), leading to greater acceptance of drug treatment plans (Babel et al., 2021) and ultimately reducing adverse drug reactions (Dsouza et al., 2025) and healthcare costs (Khanna et al., 2022).

This study presents an ML-based warning model t designed to identify key intervention points in the medication management process and optimize the pharmacist-led prescription review system. The aim is to evaluate the effectiveness of the model in improving prescription appropriateness for surgical patients, reducing adverse drug events (ADEs), and shortening hospital stays. By focusing on surgical patients, the study seeks to contribute to the standardization of clinical medication management, thereby enhancing patient safety and improving healthcare outcomes in perioperative care.

## 2 Methods

### 2.1 Design

**FIGURE 1:**
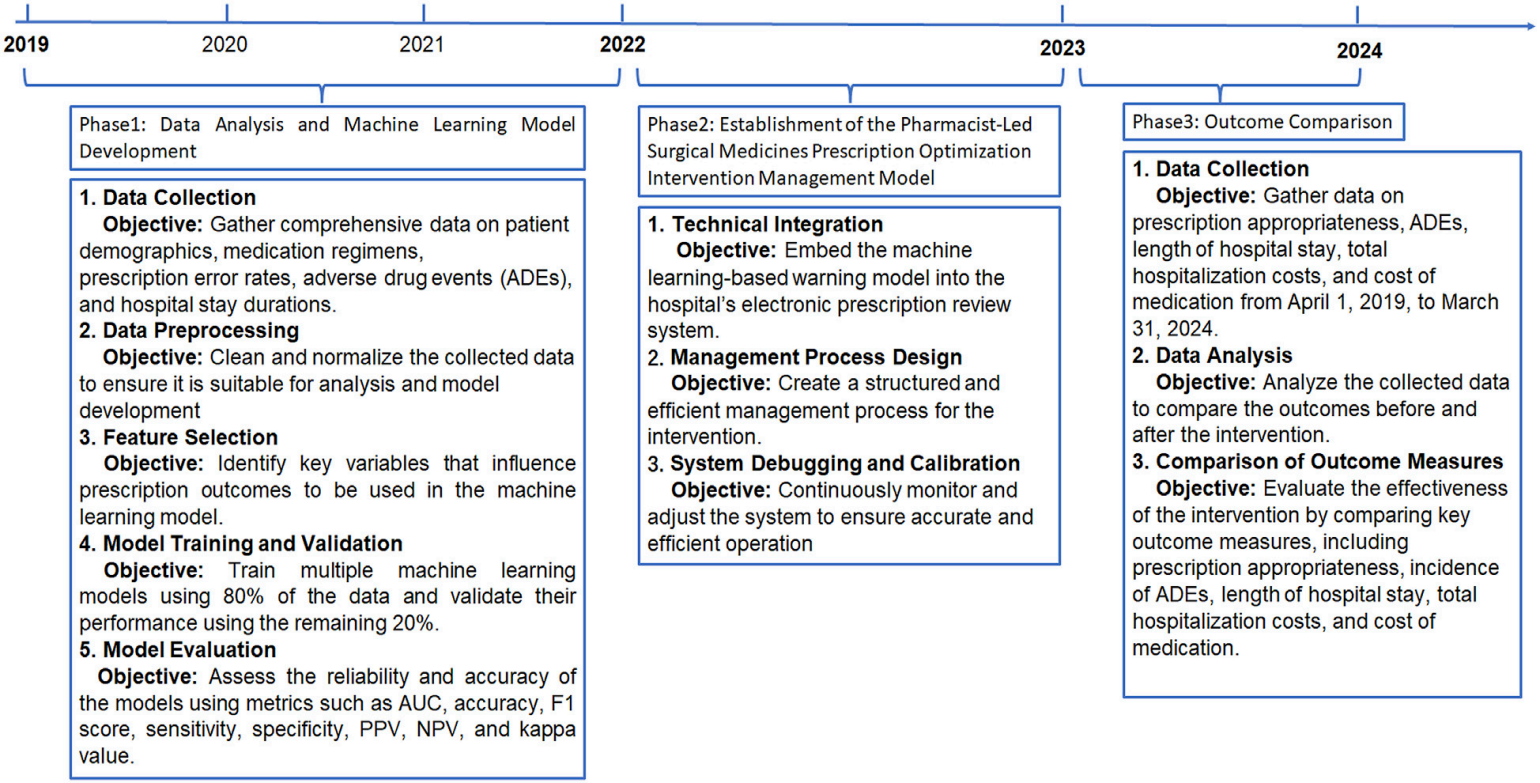
Flowchart. ADRs: Adverse Drug Reactions; AUC: Area Under the ROC Curve; PPV: positive predictive value; NPV: negative predictive value; PPEs: potential prescription errors.

This study employed a retrospective cohort design combined with a prospective implementation phase to evaluate the effectiveness of a pharmacist-led prescription optimization intervention service for surgical patients. The study was structured into three phases: data analysis and ML model development, establishment of a pharmacist-led management model, and outcome evaluation (Figure 1). This study was approved by the Ethics Committee of our hospital (Ethics Number: 2021-K-K229).

### 2.2 Setting and participants

The study was conducted at a tertiary hospital, which was the first to implement an electronic system-assisted review service for all inpatient prescriptions (beginning on 1 April 2019). The participant cohort included surgical patients admitted between April 2019 and March 2024. Patients were selected based on the following criteria: (1) Patients underwent surgery during hospitalization; (2) Patients were aged 18 years or older; **(3)** Patients had multiple comorbidities (defined as having at least two chronic conditions) (Skou et al., 2022) and complex medication regimens (defined as the use of five or more medications during hospitalization) (Pazan and Wehling, 2021). Exclusion criteria included: (1) Patients with incomplete medical records; (2) Patients transferred from other hospitals.

### 2.3 Data collection

Data was collected from the electronic prescription review (EPR) system. Key data points included: patient demographics (gender, age, number of comorbidities, length of stay, incidence of ADRs, total hospitalization costs, cost of medication); surgical-related information (type of surgery, days from hospitalization to surgery, and level of surgery complexity); prescription-related information (type of prescription, number of concurrent medications, type of medication, route of administration, grade of prescription, and type of PPEs); and intervention Information (prescribers’ seniority, prescribers’ gender, and pharmacist training).

### 2.4 Definition and evaluation of PPEs

To define Potential Prescription Errors (PPEs), we utilized a set of criteria embedded in the commercialization EPR system, which has been implemented in multiple leading hospitals across China. These criteria were developed based on authoritative literature, clinical guidelines, drug package inserts, and national drug use policies.

Before the implementation of EPR system, our hospital established a Prescription Review Rules Panel comprising multidisciplinary experts, including clinical pharmacists, prescribers, and other healthcare professionals. The panel developed the criteria using established resources such as the Beers Criteria (American Geriatrics Society, 2023), STOPP/START Criteria (O'Mahony et al., 2015), Centers for Disease Control and Prevention (CDC) opioid prescribing guidelines (Dowell et al., 2022), and other relevant guidelines.

After updating the criteria based on these resources, the panel finalized them through two rounds of the Delphi method. The updated rules were updated with monthly to address evolving clinical needs.

The included rules were embedded in the hospital’s EPR system, which contained approximately 90,000 rules. To facilitate the implementation, prescriptions were categorized into eight grades according to these rules: (1) Grades 0–4: Defined as appropriate prescriptions. Based on existing evidence, these prescriptions were likely to benefit the patient. (2) Grades 5–6: Defined as potential errors prescription. These prescriptions required further evaluation by a pharmacist, as the benefits and risks could not be conclusively determined from existing evidence. (3) Grade 7: Defined as error prescriptions. Based on existing evidence, these prescriptions had clear contraindications or risks for the patient.

According to the prescription review process, grades 5–6 prescriptions were forwarded to the pharmacist’s interface for further review. If the prescription was considered as appropriate by the pharmacist, it could proceed to dispensing; if considered a PPE, it was sent back to the prescriber’s interface for reconsideration. The prescriber then decided whether to continue with the prescription based on the pharmacist’s suggestion. Due to disagreements between pharmacists and prescribers in resolving PPEs identified as grades 5–6, the communication and acceptance process required considerable effort, making it challenging to efficiently manage risks for these prescriptions. Therefore, grades 5–6 prescriptions were included as the subject of investigation in this study. Additionally grades 5–6 PPEs were defined according to the following criteria: (1) Contraindicated drug; (2) Drug interactions; (3) Incorrect dilution; (4) Incompatibilities; (5) Incorrect infusion rate or order; (6) Non-compliance with hospital policies; (7) Incorrect duration of treatment; (8) Wrong utilization.

### 2.5 Study phases

This study was conducted in three phases to optimize and evaluate the SMPOP service model. The phases include data analysis and ML model development, establishment of the SMPOP intervention model, and outcome evaluation. Figure 1 summarizes the key activities and timeline of each phase.

### 2.6 Phase 1: Data analysis and ML model development (April 1, 2019 to 31 March 2022)

#### 2.6.1 Data Variables

The data was divided into an accepted group and an unaccepted group based on the prescribers’ decisions which were informed by the pharmacist’s opinion. Independent variables included patient variables (gender, age, number of comorbidities); surgical variables (type of surgery, days from hospitalization to surgery, level of surgery complexity); prescription variables (type of prescription, number of concurrent medications, type of medication, route of administration, grade of prescription, and type of PPEs); and intervention variables (prescriber’s seniority, prescriber’s gender, pharmacist’s training).

#### 2.6.2 Model Development

The 6,983 prescriptions were split into a training set and an internal validation set in a ratio of 8:2. The 15 aforementioned features were used to develop the prediction models. 11 ML models were used to predict whether the prescriber accepts the pharmacist’s decision: Naive Bayes (NB), RF, extreme gradient boosting (XGBoost), artificial neural network (ANN), SVM, decision tree (DT), extra trees (ET), gradient boosting machine (GBM), light gradient boosting machine (LightGBM), logistic regression (LR), and adaptive boosting (AdaBoost).

#### 2.6.3 Model Evaluation

The reliability of the models was assessed using common evaluation metrics (Mandrekar, 2010; McHugh, 2012), including the area under the receiver operating characteristic curve (AUC), accuracy, F1 score, sensitivity, specificity, positive predictive value (PPV), negative predictive value (NPV), and kappa coefficient. The prediction models were validated using Five-fold and Ten-fold cross-validation.

#### 2.6.4 Feature Selection and Model Interpretation

SHapley Additive exPlanations (SHAP) was used to interpret the models and estimate the contribution of each variable (Hu et al., 2024). SHAP values were used to reduce the features from 15 to the top three based on feature importance ranking. The Delong nonparametric method and binomial exact test in MedCalc (version 22.030) (DeLong et al., 1988) were used to compare the differences in AUC for different features.

### 2.7 Phase 2: Establishment of the pharmacist-led surgical Medicines prescription optimization intervention management model (April 1, 2022 to 31 March 2023)

**FIGURE 2:**
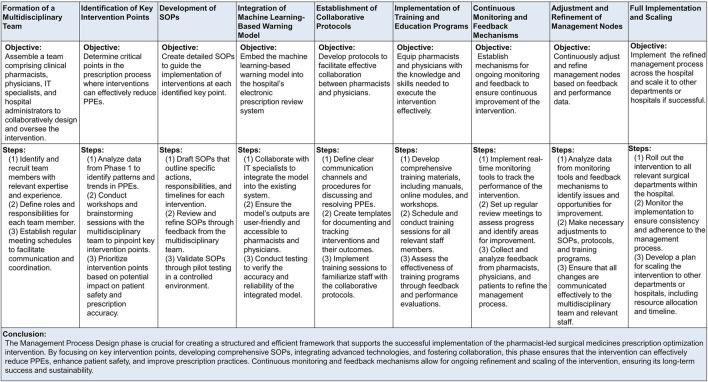
Establishment and Implementation of the Pharmacist-Led Surgical Medicines Prescription Optimization and Prediction (SMPOP)^®^ Service Model. PPEs: potential prescription errors; SOP: standard operating procedures.

In this phase, version 1.0 of a pharmacist-led Surgical Medicines Prescription Optimization and Prediction (SMPOP) ^®^ service model was developed. Activities included: (1) Technical Integration: The ML-based optimization and prediction model was embedded into the hospital’s EPR system. (2) System Debugging and Calibration: Continuous monitoring and adjustment of the system were performed made to ensure the system operated accurately and efficiently operation. (3) SMPOP Process Design: Based on significant findings from Phase 1, multiple management nodes were established, and the overall process clearly defined (Figure 2).

### 2.8 Phase 3: Outcome evaluation (April 1, 2023 to 31 March 2024)

During this phase, the effectiveness of the SMPOP service was assessed. Data from the following periods were collected and analyzed to measure the impact of the SMPOP service on patient outcomes: (1) April 2019 to March 2020 (ML Development Period): This period was dedicated to developing ML models using data from the derivation cohort. (2) April 2020 to March 2021 (External Validation Period): During this period, the developed models were externally validated using a separate dataset to ensure robustness and generalizability. (3) April 2023 to March 2024 (Post-Implementation Evaluation Period): This period involved evaluating patient outcomes after the implementation and stabilization of the pharmacist-led prescription optimization intervention service model.

The following key outcome measures were evaluated: (1) Prescription appropriateness: Evaluated based on the prediction of PPEs and the acceptance rate of pharmacist recommendations by prescribers. (2) ADRs: Measured by the total incidence rate of ADRs across the three periods to determine the effectiveness of the intervention in reducing medication-related harm. (3) Length of Hospital Stay: A comparison of the average duration of hospital stays across the three periods was used to highlight any improvements in patient recovery times and hospital efficiency. (4) Total Hospitalization Costs: Analyzed by comparing the total costs incurred during hospital stays across the three periods, which includes all expenses related to patient care and treatment. (5) Cost of Medication: Evaluated by comparing the total expenditure on medications across the three periods, reflecting the cost-effectiveness of the optimized prescription practices.

### 2.9 Statistical analysis

Statistical analyses were performed using Python (Spyder-py3), IBM SPSS Statistics (version 25.0), and R (version 4.4.0). Decision curve analysis (DCA) was performed using R (version 4.4.0), while precision-recall curve analysis (P-R curve analysis) was performed using Python (Spyder-py3). In the comparative analysis between the two groups, categorical variables were expressed as frequencies (percentages) and compared using the chi-square test. For normally distributed continuous data, descriptive statistics were presented as mean ± standard deviation, and the independent sample t-test was used. For non-normally distributed continuous data, distribution characteristics were described using the interquartile range (IQR), and the Mann-Whitney U test was applied. A *p*-value <0.05 was considered statistically significant.

## 3 Result

### 3.1 Descriptive statistics of the number of each prescription

A total of 2,049,499 prescriptions were issued for both the pre- and post-implementation SMPOP service cohorts between 1 April 2019, and 31 March 2024. For pre-implementation cohorts, 793,395 prescriptions were classified as grades 5 and 6 by the system and were sent to the pharmacist interface for further review. Among these, 6,983 prescriptions were deemed to have PPEs and were included for ML model development during Phase 1. Prescriptions for external validation were included in Phase 1. In Phase 3, the number of prescriptions included was 3,307.

4,208 patients were associated with the 6,983 prescriptions deemed to have PPEs: 62.55% of patients had one prescription, 22.81% had two prescriptions, 7.87% had three prescriptions, and 6.86% of patients had more than three prescriptions (Supplementary Table S1).

### 3.2 Characteristics of patients and surgeries

For the analysis of the ML model development, of the 6,983 PPE prescriptions,43.90% were accepted by prescribers and defined as the ‘accepted group’, while 56.10% were not accepted and were categorized as the ‘unaccepted group’. Comparison between the two groups indicated that the type of surgery, days from hospitalization to surgery, level of surgery complexity, type of prescription, number of concurrent medications, type of medication, route of administration, grade of prescription, type of PPEs, prescriber’s gender, and pharmacist training were significantly different (*p* < 0.05). Specifically, regarding the grade of prescription, prescriptions of grade 5 accounted for 84.52% of all PPE prescriptions, and made up 83.33% of the ‘unaccepted’ group (*p* = 0.001). For all types of PPE, the most common error was inappropriate indication (67.76%), accounting for 68.30% of the ‘unaccepted’ group (*p* < 0.001). Others, such as gender, age, number of comorbidities, or prescriber’s seniority, showed no significant statistical differences (*p* > 0.05) (Supplementary Table S2). A comparison of demographics and variables between the training set, internal validation set, and external validation set was provided in Supplementary Table S3.

## 4 ML model development

### 4.1 Model development and performance comparison

**FIGURE 3:**
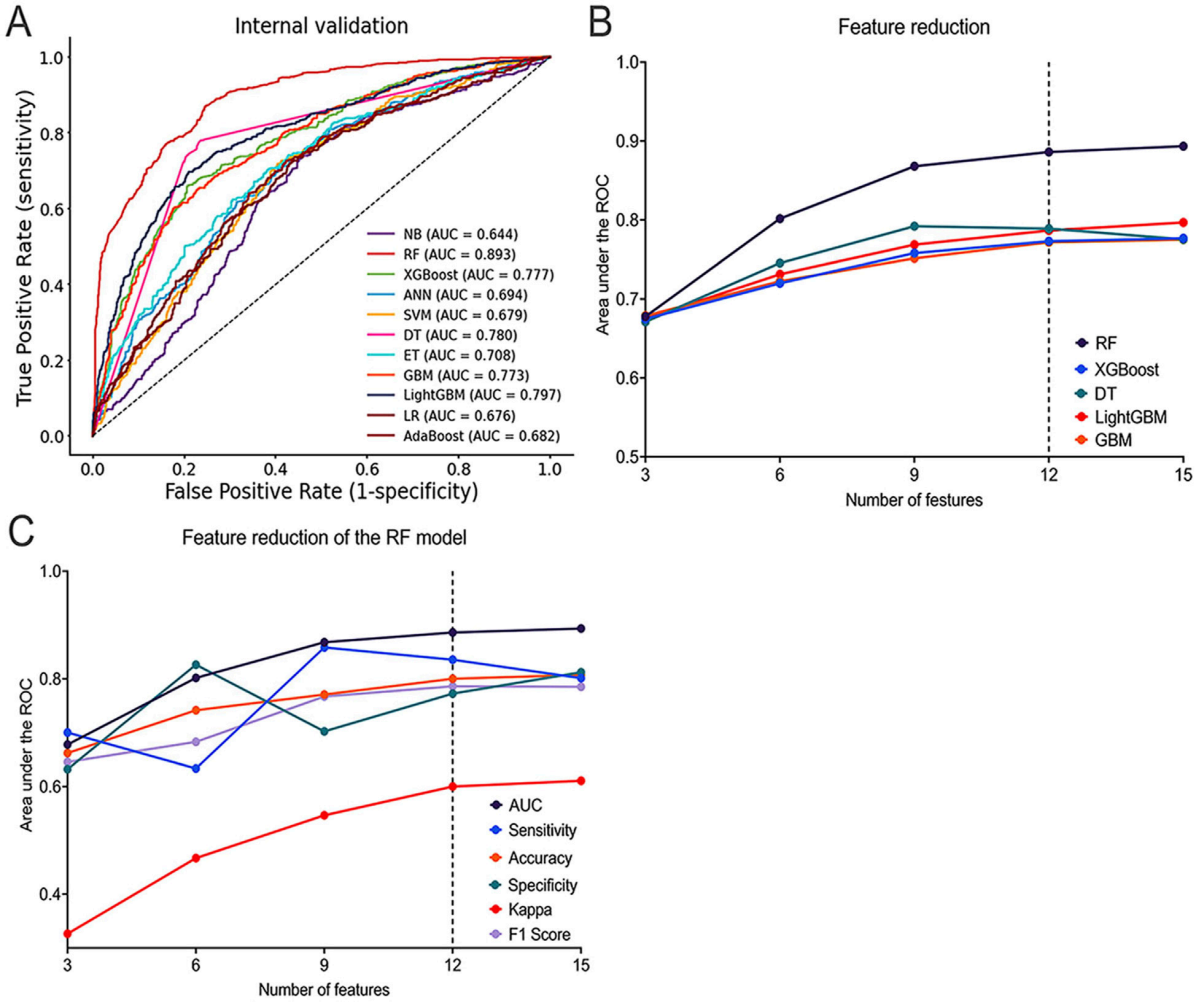
Performance of ML Models in Predicting Whether the prescriber accepts the pharmacist’s decision. **(A)** ROC curves of the top five best-performing ML models; **(B)** AUC of the top five best-performing ML models with different numbers of features; **(C)** AUC, Sensitivity, Accuracy, Specificity, Kappa, and F1 Score of the RF model with different numbers of features. ROC: Receiver Operating Characteristic; AUC: Area Under the ROC Curve; AdaBoost: Adaptive Boosting; ANN: Artificial Neural Network; DT: Decision Tree; ET: Extra Trees; GBM: Gradient Boosting Machine; LightGBM: Light Gradient Boosting Machine; LR: Logistic Regression; ML: Machine Learning; RF: Random Forest; SVM: Support Vector Machine; XGBoost: Extreme Gradient Boosting.

During Phase 1, 11 ML models were generated to predict whether the prescriber accepts the pharmacist’s recommendation. Among the 11 ML models, the RF model had the best predictive performance in terms of AUC (AUC = 0.893), followed by the LightGBM model (AUC = 0.797), DT model (AUC = 0.780), XGBoost model (AUC = 0.777), and GBM model (AUC = 0.773) (Figure 3A). For other performance metrics, these 5 ML models also performed better than the other six models (Supplementary Table S4), and were therefore selected as further analysis models.

In the SHAP summary plot for all features, the ‘type of prescription’ consistently ranked as the most important feature across the five optimal ML models (Supplementary Figure S1). During the feature reduction using feature engineering, changes in the AUC values indicated that the RF model demonstrated the best predictive capability (Figure 3B). Additionally, performance evaluations of the RF model with varying numbers of features further confirmed its strong predictive capability (Figure 3C; Supplementary Table S5).

### 4.2 Final model selection

The final model was selected through the feature reduction process of the RF model. The DeLong and binomial exact tests were used to compare the differences in AUC with different features in predicting whether the prescriber accepts the pharmacist’s decision. The results showed that the 15-feature model (AUC = 0.893, 95%CI = 0.876–0.909) was significantly better than the 9-feature model (AUC = 0.868, ΔAUC = 0.025, *p* < 0.001, 95% CI = 0.849–0.885), the 6-feature model (AUC = 0.802, ΔAUC = 0.092, *p* < 0.001, 95% CI = 0.780–0.822), and the 3-feature model (AUC = 0.678, ΔAUC = 0.216, *p* < 0.001, 95% CI = 0.653–0.702) but was not significantly better than the 12-feature model (AUC = 0.886, ΔAUC = 0.007, *p* = 0.092, 95%CI = 0.868–0.902). This implies that reducing the number of features did not significantly affect model performance (Supplementary Figure S2A; Supplementary Table S6).

The DCA curve was used to compare prediction models with varying numbers of features. Results showed that the 12-feature model provided a greater net benefit, demonstrating strong predictive performance across different thresholds (Supplementary Figure S2B). Additionally, the P-R curve revealed that the 12-feature model (PR-AUC = 0.867) performed slightly below the 15-feature model (PR-AUC = 0.874), indicating that both models offer similarly high clinical utility (Supplementary Figure S2C).

Finally, the 12-feature RF model (including gender, age, number of comorbidities, type of surgery, level of surgery complexity, days from hospitalization to surgery, type of prescription, number of concurrent medications, type of medication, route of administration, type of PPEs, prescriber’s seniority) was selected as the final model for further analysis. The final RF model for predicting prescriber opinion had an AUC of 0.886, a sensitivity of 0.836, a specificity of 0.773, PPV of 0.742, NPV of 0.857, accuracy of 0.800, F1 score of 0.786, and kappa of 0.600.

To validate the adequacy of the sample size and the robustness of the model, additional cross-validation was performed. The final RF model achieved a mean AUC of 0.891 ± 0.012 in five-fold cross-validation (Supplementary Figure S3A) and 0.898 ± 0.014 in ten-fold cross-validation (Supplementary Figure S3B). Additionally, the final model had a mean AUC of 0.912 ± 0.026 for 2019 (n = 2,734) and 0.885 ± 0.012 for 2020 (n = 4,249) according to ten-fold cross-validation (Supplementary Table S7).

### 4.3 External validation of the final model

Data from 2021 to 2022 were used as external validation data. The RF model performed well in both internal and external validation. Internal validation showed an AUC of 0.886, while external validation showed an AUC of 0.786, with a ΔAUC of 0.100, indicating the model’s consistency and generalizability across different datasets (Supplementary Figure S4). Despite a slight decrease in the AUC during external validation, other key performance metrics, such as accuracy, precision, PPV, NPV, and specificity, remained high on the external dataset, which demonstrated the model’s effectiveness and robustness for new data (Supplementary Table S8).

### 4.4 Model explanation

**FIGURE 4:**
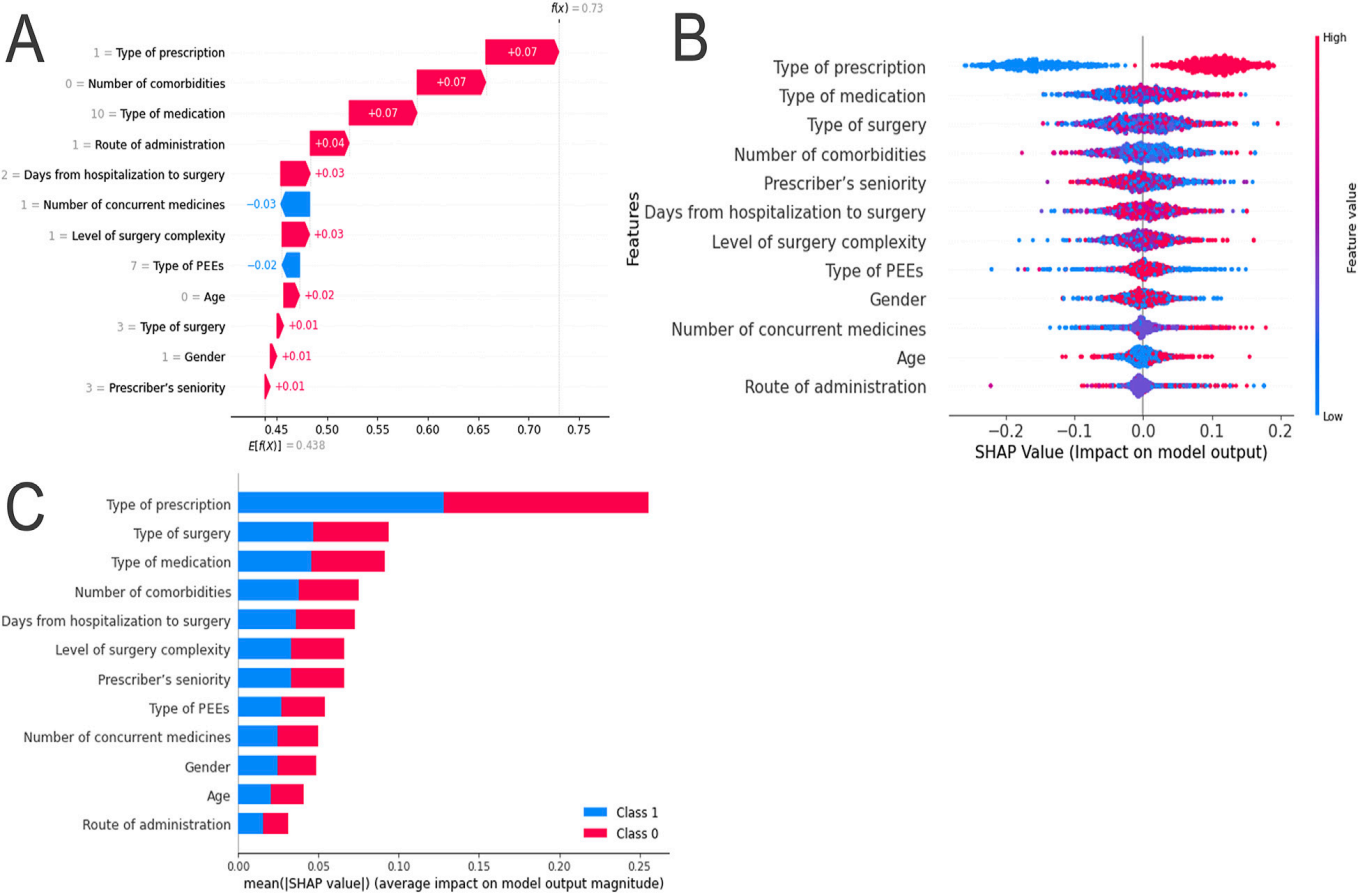
SHAP Explanation of the RF Model. **(A)** SHAP Force Plot: Contribution of each feature value for a single sample; **(B)** SHAP Summary Plot: Summary of SHAP values for each variable; **(C)** SHAP Summary Bar Plot: Absolute mean of SHAP values for each variable.

Using the SHAP method, both local and global interpretations of the model were provided. Local interpretation was used to understand the specific prediction process for individual samples in the RF model. Variables such as gender, age, number of comorbidities, type of surgery, level of surgery complexity, days from hospitalization to surgery, type of prescription, type of medication, route of administration, and prescriber’s seniority were found to have a positive impact on the SHAP values. In contrast, the number of concurrent medications and type of PPEs were found to have a negative impact on the SHAP values (Figure 4A).

Global interpretation was used to describe the overall functionality of the model. By calculating and plotting the SHAP values for each feature across all samples, the SHAP values for each sample were summarized and variables ranked in descending order. For example, the SHAP values for long-term prescriptions were found to be relatively low, indicating a significant negative impact on model predictions (Figure 4B). The importance of the features was evaluated based on the average absolute SHAP values, which were arranged in descending order. According to the RF model, the five most important features were identified as type of prescription, type of surgery, type of medication, number of comorbidities, and days from hospitalization to surgery (Figure 4C). SHAP dependence plots were used to understand how individual features influenced the model’s output. The relationships between the actual values and SHAP values for these 12 features are illustrated in Supplementary Figure S5.

## 5 Outcome evaluation

The changes in prescribers' acceptance of pharmacists' recommendations, length of stay, total hospitalization costs, cost of medication, and number of ADRs were compared across three periods to evaluate the effectiveness of the SMPOP service. An increase in the acceptance rate of pharmacists' recommendations was observed among the three groups, along with decreases in the length of stay, total hospitalization costs, cost of medication, and ADRs (Table 1). Compared to the ML development period and external validation period, the acceptance rate of pharmacists’ recommendations in the post-implementation evaluation period increased from 40.63% to 71.30%, the length of stay was reduced from 13 days (IQR: 8, 22) (*P* < 0.001) to 7 days (IQR: 9, 15) (*p* < 0.001), total hospitalization costs decreased from 32,263.42 RMB (IQR: 16,880.46,68,921.29) (*p* < 0.001) to 20,842.89 RMB (IQR: 13,516.59, 32,055.51) (*p* < 0.001), cost of medication decreased from 7847.67 RMB (IQR: 3815.45,19,980.16) (*p* < 0.001) to 4,712.92 RMB (IQR: 2866.63, 7716.55) (*p* < 0.001), and the ADR rate was reduced from 0.4% to 0.18% (*p* = 0.095).

**TABLE 1:** Evaluation of Outcomes during the different period.

Variable	ML development period (N = 6983)	External validation period (N = 3495)	Post-implementation evaluation period (N = 3307)	*p*-value
The acceptance rate of pharmacist recommendations by prescribers, (n, %)				<0.001
Accepted group	3069 (43.95)	1420 (40.63)	2358 (71.30)	
Unaccepted group	3914 (56.05)	2075 (59.37)	949 (28.70)	
Length of stay, (median, IQR)	15 [9,24]	13 [8,22]	7 [9,15]	<0.001
Total hospitalization costs, (median, IQR)	36160.11 [19550.97-71673.10]	32263.42 [16880.46,68921.29]	20842.89 [13516.59,32055.51]	<0.001
Medication costs, (median, IQR)	8794.98 [4162.56–19805.44]	7847.67 [3815.45,19,980.16]	4712.92 [2866.63,7716.55]	<0.001
ADRs (n, %)	13 (0.19)	14 (0.4)	6 (0.18)	0.095

Note: P < 0.05 is considered statistically significant; IQR: interquartile range; ADRs: adverse drug reaction.

## 6 Discussion

This study demonstrated the potential of an ML-based model integrated into a pharmacist-led SMPOP service to enhance prescription practices for surgical patients. Given the complexity of surgical medication regimens, optimizing prescriptions was essential to improving outcomes and reducing costs. Using a three-phase approach, namely, data analysis and model development, management model establishment, and outcome evaluation, the study showed how predictive analytics can enhance the identification and resolution of PPEs. The RF model proved most effective in predicting the acceptance of pharmacist recommendations, enhancing prescription appropriateness, reducing ADRs, shortening hospital stays, and saving costs. These findings highlight the benefits of integrating ML with clinical decision-making, providing a framework for better medication management and patient safety in surgical care.

A significant proportion of identified PPEs remained unaccepted by prescribers from 2019 to 2022. Similar to findings from prior research, a study from the United States found that around 30% of discovered medication errors reached patients (Sacks et al., 2009). However, that investigation did not focus on surgical settings, a factor which may have influenced how unaccepted PPEs were resolved. Even with pharmacy-based interventions, 53% of drug errors during the administration stage persisted (FitzHenry et al., 2007), highlighting the difficulty of comparing PPE rates across studies, as they can vary based on context, methodology, and definitions of prescription errors (Lisby et al., 2010; Saif et al., 2024; Alsabri et al., 2024). Consequently, these comparison data should be interpreted cautiously. Nonetheless, the high proportion of unresolved PPEs underscores the need to enhance efforts to address these issues.

The final ML prediction model included critical features: age, gender, number of comorbidities, type of surgery, level of surgical complexity, days from hospitalization to surgery, number of concurrent medications, type of medication, route of administration, type of PPEs, prescriber’s seniority, and type of prescription. Inclusion of these features could enhance the efficiency of resolving unaccepted PPEs and provide a nuanced understanding of effective strategies (Johns et al., 2024; Mahomedradja et al., 2023; Tansuwannarat et al., 2023). Our study identified that male patients with comorbidities undergoing complex surgeries were at higher risk for unresolved PPEs, aligning with prior research indicating that surgical complexity and comorbidities are significant predictors, often exacerbating communication challenges in resolving PPEs (Tonelli et al., 2018; Nanji et al., 2016).

While gender had not traditionally been viewed as a significant factor, our findings suggested that prescriptions for male patients required closer collaboration between prescribers and pharmacists. Medications administered *via* non-oral routes, such as intravenous, intramuscular, subcutaneous, and *via* inhalation, were flagged as high-risk categories, indicating that non-oral routes were associated with higher rates of PPEs due to their complexities (Alsabri et al., 2024). Additionally, our study found that junior pharmacists were more likely to reduce unresolved PPEs, contradicting the notion that experience necessarily enhances PPE resolution (Jaam et al., 2021; Dean et al., 2002). This may be due to junior pharmacists being more attuned to newer protocols and dedicating more time to PPE-related issues, supporting research advocating for fresh perspectives and up-to-date training in the healthcare workforce (Newby et al., 2019; Gillani et al., 2021).

In this study, the RF model was selected for its superior predictive performance in identifying PPEs and supporting pharmacist-led interventions. Among the tested ML models, RF achieved the highest AUC, making it optimal in this context. This finding aligns with prior studies highlighting the effectiveness of RF models in predicting clinical outcomes by managing complex, non-linear interactions among variables (Hu et al., 2024). Unlike LR and SVM commonly used in earlier prescription optimization studies (Chalasani et al., 2023; Couronné et al., 2018; Marchi et al., 2022), RF showed superior adaptability to patient, surgical, and prescription-related factors (Hassan et al., 2023). Furthermore, feature reduction confirmed the RF’s ability to maintain high predictive accuracy with fewer variables. The 12-feature model selected through SHAP analysis maintained strong performance comparable to the full 15-feature model, as confirmed by DCA, PR-AUC, and statistical tests. The RF model’s capacity to handle large feature sets and provide interpretable results through SHAP aligns with the complexities of clinical decision-making in surgical settings.

The SMPOP service integrated an ML-based warning system into the EPR process, improving the identification of high-risk prescriptions. Predictive analytics established key intervention nodes, facilitating collaboration between pharmacists and the healthcare team. This framework was implemented and calibrated through continuous feedback, ensuring alignment with clinical practices and enhancing its utility in optimizing surgical medication use. In this study, the RF within SMPOP demonstrated strong predictive accuracy and efficiency, even after feature reduction that integrated patient factors, surgical complexities, prescription details, and both pharmacist and prescriber characteristics. Traditional pharmacist-led interventions, though effective, often faced limitations in comprehensiveness and scalability (Jaam et al., 2021; Li et al., 2024; Moon et al., 2020; Mostafa et al., 2020) This integrated approach contrasts with earlier models that focused on fewer variables or lacked a holistic view of factors affecting prescription appropriateness (Bates et al., 2021; Alotaibi and Federico, 2017).

ML model have demonstrated significant advantages in detecting inappropriate prescriptions and identifying factors leading to prescription errors, as confirmed by studies conducted by Johns et al. (2024), Mahomedradja et al. (2023). After the introduction of ML model, this study shows that the acceptance rate of pharmacists' recommendations increased from 43.9% to 71.3%, indicating that the model enhanced the scientific rigor and credibility of pharmacists' advice. In terms of types of medication errors, this study found that “Wrong utilisation” accounted for 67.76%, being the most common type. Similarly, a 10-year retrospective analysis also indicates that dosage and drug selection errors are the most frequent types of errors (Tansuwannarat et al., 2023). In short, by combining ML models with pharmacist-led intervention services, this study further improves the effectiveness of prescription optimization.

## 7 Conclusion and limitations

This study demonstrates the effectiveness of an ML-based pharmacist-led prescription intervention in optimizing prescriptions, improving appropriateness, reducing ADEs, shortening hospital stays, and lowering healthcare costs. By integrating advanced technologies with medication management expertise, this approach offers a promising strategy for improving patient outcomes and underscores the importance of continuous pharmacist-led innovation in medication safety.

However, because this study was conducted in a single tertiary hospital, its findings may have limited generalizability to other healthcare settings. Future research should explore the intervention’s applicability across diverse hospital environments and patient populations, further refine the ML models to improve predictive accuracy, identify additional features, and evaluate the intervention’s long-term impacts on clinical outcomes.

## Data Availability

The raw data supporting the conclusions of this article will be made available by the authors, upon reasonable request. Requests to access the datasets should be directed to shangnan@vip.163.com.
