# Supplementary material for: Pharmacist-led surgical medicines prescription optimization and prediction service improves patient outcomes - a machine learning based study
Source: Front Pharmacol. 2025 Mar 14;16:1534552. doi: 10.3389/fphar.2025.1534552 (PMC11949800; doi:10.3389/fphar.2025.1534552)
Supplement: Supplementary file 1 [file DataSheet1.docx]

**Supplemental Material**

**List of Supplemental Table materials:**

**Supplemental Table 1**: Distribution of the Number of Prescriptions for Surgical Patients (*Page 2*)

**Supplemental Table 2**: Comparison of Demographic and Clinical Characteristics and Outcomes Between Whether the Prescriber Accepts the Pharmacist's Decision in the 2019-2020 Derivation Cohort. *(Page 3-5)*

**Supplemental Table 3:** Comparison of demographic and variable characteristics between training, internal validation, and external validation datasets. *(Page 6-8)*

**Supplemental Table 4:** Performance of the ML models for whether the physician accepts the pharmacist's decision. (*Page 9*)

**Supplemental Table 5:** Performance of the RF model with varied numbers of features for whether the physician accepts the pharmacist's decision. (*Page 10*)

**Supplemental Table 6:** Delong comparison of different features under the RF model with 95% confidence intervals. (*Page 11*)

**Supplemental Table 7:** Performance of the final RF model for whether the physician accepts the pharmacist's decision occurred. (*Page 12*)

**Supplemental Table 8:** Comparison of metrics for internal and external validation of the RF model. (*Page 13*)

**List of Supplemental Table materials:**

**Supplemental Figure 1:** SHAP summary dot plots of all features for the top five best-performing machine learning models. (*Page 14*)

**Supplemental Figure 2**: Predictive Performance of the RF Model After Reducing Features. (*Page 15*)

**Supplemental Figure 3:** Predictive performance of the final RF model in cross-validation. (*Page 16*)

**Supplemental Figure 4**: Comparison of Internal and External Validation ROC Curves. (*Page 17*)

**Supplemental Figure 5:** SHAP dependence plots. (*Page 18*)

**Supplementary Table 1**: Distribution of the Number of Prescriptions for Surgical Patients

| Number of Prescriptions | Proportion of Patients | Number of Patients | Total Number of Prescriptions |
| --- | --- | --- | --- |
| 1 | 62.55% | 2630 | 2631 |
| 2 | 22.81% | 950 | 1918 |
| 3 | 7.87% | 331 | 993 |
| >3 | 6.85% | 286 | 1465 |

**Supplemental Table 2**: Comparison of Demographic and Clinical Characteristics and Outcomes Between Whether the Prescriber Accepts the Pharmacist's Decision in the 2019-2020 Derivation Cohort

| Variable | Accepted group  (N=3069) | Unaccepted group  (N=3914) | χ2 | *p*-value |
| --- | --- | --- | --- | --- |
| **Gender, n (%)** |  |  | 2.983 | 0.084 |
| Female | 1308(42.62) | 1749(44.69) |  |  |
| Male | 1761(57.38) | 2165(55.31) |  |  |
| **Age, n (%)** |  |  | 3.708 | 0.054 |
| ＜65 | 2137(69.63) | 2808(71.74) |  |  |
| ≥65 | 932(30.37) | 1106(28.26) |  |  |
| **Number of comorbidities, n (%)** |  |  | 8.401 | 0.078 |
| None | 1039(33.85) | 1259(32.17) |  |  |
| 1 | 1070(34.86) | 1432(36.59) |  |  |
| 2 | 510(16.62) | 632(16.15) |  |  |
| 3 | 232(7.56) | 346(8.84) |  |  |
| ≥3 | 218(7.10) | 245(6.26) |  |  |
| **Type of surgery, n (%)** |  |  | 54.605 | <0.001 |
| Biliary tract | 264(8.60) | 351(8.97) |  |  |
| Amputation | 280(9.12) | 383(9.79) |  |  |
| Genitourinary | 495(16.13) | 790(20.18) |  |  |
| Skin and deep tissue (incision, drainage, local excision, skin graft) | 552(17.99) | 696(17.78) |  |  |
| Breast | 15(0.49) | 55(1.41) |  |  |
| Hernia repair | 26(0.85) | 49(1.25) |  |  |
| Upper and lower GIT | 340(11.08) | 377(9.63) |  |  |
| Neurosurgery (craniotomy) or craniotomy | 194(6.32) | 259(6.62) |  |  |
| Head and neck | 186(6.06) | 213(5.44) |  |  |
| Cardiothoracic | 156(5.08) | 158(4.04) |  |  |
| Other | 561(18.28) | 583(14.90) |  |  |
| **Days from hospitalization to surgery, n (%)** |  |  | 11.664 | 0.009 |
| Day<2 | 525(17.11) | 661(16.89) |  |  |
| 2≤Day<4 | 487(15.87) | 724(18.50) |  |  |
| 4≤Day<8 | 889(28.97) | 1160(29.64) |  |  |
| Day≥8 | 1168(38.06) | 1369(34.98) |  |  |
| **Level of surgery complexity, n (%)** |  |  | 23.17 | <0.001 |
| 1 | 77(2.51) | 141(3.60) |  |  |
| 2 | 635(20.69) | 796(20.34) |  |  |
| 3 | 1220(39.75) | 1706(43.59) |  |  |
| ≥3 | 1137(37.05) | 1271(32.47) |  |  |
| **Type of prescription, n (%)** |  |  | 578.227 | <0.001 |
| Stat | 769(25.06) | 2097(53.58) |  |  |
| Long-term | 2300(74.94) | 1817(46.42) |  |  |
| **Number of concurrent medicines, n (%)** |  |  | 49.449 | <0.001 |
| 1 | 449(14.63) | 622(15.89) |  |  |
| 2 | 2109(68.72) | 2826(72.20) |  |  |
| 3 | 205(6.68) | 248(6.34) |  |  |
| ≥3 | 306(9.97) | 218(5.57) |  |  |
| **Type of medication, n (%)** |  |  | 76.985 | <0.001 |
| Sensory organs | 6(0.20) | 6(0.15) |  |  |
| Respiratory system | 41(1.34) | 38(0.97) |  |  |
| musculoskeletal | 310(10.10) | 456(11.65) |  |  |
| Anti-infective | 898(29.26) | 1344(34.34) |  |  |
| antineoplastic and immunomodulating | 43(1.40) | 85(2.17) |  |  |
| Genitourinary system and sex hormones | 18(0.59) | 34(0.87) |  |  |
| Nervous system | 221(7.20) | 299(7.64) |  |  |
| Endocrine system | 56(1.82) | 53(1.35) |  |  |
| Digestive system and metabolism | 984(32.06) | 1019(26.03) |  |  |
| Cardiovascular system | 162(5.28) | 166(4.24) |  |  |
| Blood and hematopoietic system | 193(6.29) | 172(4.39) |  |  |
| Others | 137(4.46) | 242(6.18) |  |  |
| **Route of administration, n (%)** |  |  | 50.535 | <0.001 |
| Oral | 332(10.82) | 326(8.33) |  |  |
| Iv/Im/Sc | 2434(79.31) | 3283(83.88) |  |  |
| Inhalers | 7(0.23) | 41(1.05) |  |  |
| Other | 296(9.64) | 264(6.75) |  |  |
| **Grade of prescription, n (%)** |  |  | 10.277 | 0.001 |
| 5 | 2642(86.09) | 3260(83.29) |  |  |
| 6 | 427(13.91) | 654(16.71) |  |  |
| **Type of PPEs, n (%)** |  |  | 68.592 | <0.001 |
| Contra-indicated drug | 76(2.48) | 147(3.76) |  |  |
| Drug interactions | 27(0.88) | 82(2.10) |  |  |
| Inappropriate dilution | 711(23.17) | 690(17.63) |  |  |
| Incompatibilities | 51(1.66) | 112(2.86) |  |  |
| Incorrect infusion rate or order | 45(1.47) | 53(1.35) |  |  |
| Non-compliance with hospital policies | 30(0.98) | 31(0.79) |  |  |
| Wrong duration of treatment | 70(2.28) | 126(3.22) |  |  |
| **Wrong utilisation** | **2059(67.09)** | **2673(68.29)** |  |  |
| **Prescriber’s seniority, n (%)** |  |  | 3.917 | 0.141 |
| Junior | 1110(36.17) | 1367(34.93) |  |  |
| Intermediate | 873(28.45) | 1072(27.39) |  |  |
| Senior | 1086(35.39) | 1475(37.69) |  |  |
| **Prescriber’s gender, n (%)** |  |  | 5.154 | 0.023 |
| Female | 778(25.35) | 1087(27.77) |  |  |
| Male | 2291(74.65) | 2827(72.23) |  |  |
| **Pharmacist training, n (%)** |  |  | 5.456 | 0.019 |
| Yes | 2608(84.98) | 3403(86.94) |  |  |
| No | 461(15.02) | 511(13.06) |  |  |

Note: *p* < 0.05 is considered statistically significant. Upper and lower GIT: Upper and lower gastrointestinal tract; Iv/Im/Sc: intravenous, intramuscular, or subcutaneous injections; PPEs: potential prescription errors.

**Supplemental Table 3:** Comparison of demographic and variable characteristics between training, internal validation, and external validation datasets.

| Variable | Training | Internal validation | *p*-value | Derivation | External validation | *p*-value |
| --- | --- | --- | --- | --- | --- | --- |
|  | (N=5586) | (N=1397) |  | (N=6983^[a]^) | (N=3495) |  |
| **Whether the physician accepts the pharmacist's decision** |  |  | 0.858 |  |  | <0.001 |
| Accepted group | 2458(44.00) | 611(43.74) |  | 3069(43.95) | 747(21.37) |  |
| Unaccepted group | 3128(56.00) | 786(56.26) |  | 3914(56.05) | 2748(78.63) |  |
| **Gender, n (%)** |  |  | 0.289 |  |  | 0.036 |
| Female | 2463(44.09) | 594(42.52) |  | 3057(43.78) | 1455(41.63) |  |
| Male | 3123(55.91) | 803(57.48) |  | 3926(56.22) | 2040(58.37) |  |
| **Age, n (%)** |  |  | 0.679 |  |  | <0.001 |
| < 65 | 3962(70.93) | 983(70.37) |  | 4945(70.81) | 2344(67.07) |  |
| ≥ 65 | 1624(29.07) | 414(29.63) |  | 2038(29.19) | 1151(32.93) |  |
| **Number of comorbidities, n (%)** |  |  | 0.806 |  |  | <0.001 |
| None | 1825(32.67) | 473(33.86) |  | 2298(32.91) | 836(23.92) |  |
| 1 | 2013(36.04) | 489(35.00) |  | 2502(35.83) | 1250(35.77) |  |
| 2 | 922(16.51) | 220(15.75) |  | 1142(16.35) | 669(19.14) |  |
| 3 | 461(8.25) | 117(8.38) |  | 578(8.28) | 312(8.93) |  |
| ≥ 3 | 365(6.53) | 98(7.02) |  | 463(6.63) | 428(12.25) |  |
| **Type of surgery, n (%)** |  |  | 0.349 |  |  | <0.001 |
| Biliary tract | 496(8.88) | 119(8.52) |  | 615(8.81) | 286(8.18) |  |
| Amputation | 553(9.90) | 110(7.87) |  | 663(9.49) | 176(5.04) |  |
| Genitourinary | 1017(18.21) | 268(19.18) |  | 1285(18.40) | 599(17.14) |  |
| Skin and deep tissue (incision, drainage, local excision, skin graft) | 1001(17.92) | 247(17.68) |  | 1248(17.87) | 510(14.59) |  |
| Breast | 51(0.91) | 19(1.36) |  | 70(1.00) | 17(0.49) |  |
| Hernia repair | 61(1.09) | 14(1.00) |  | 75(1.07) | 10(0.29) |  |
| Upper and lower GIT | 559(10.01) | 158(11.31) |  | 717(10.27) | 460(13.16) |  |
| Neurosurgery (craniotomy) or craniotomy | 367(6.57) | 86(6.16) |  | 453(6.49) | 243(6.95) |  |
| Head and neck | 316(5.66) | 83(5.94) |  | 399(5.71) | 167(4.78) |  |
| Cardiothoracic | 245(4.39) | 69(4.94) |  | 314(4.50) | 180(5.15) |  |
| Others | 920(16.47) | 224(16.03) |  | 1144(16.38) | 847(24.23) |  |
| **Days from hospitalization to surgery, n (%)** |  |  | 0.532 |  |  | <0.001 |
| Day < 2 | 966(17.29) | 220(15.75) |  | 1186(16.98) | 615(17.60) |  |
| 2 ≤ Day < 4 | 972(17.40) | 239(17.11) |  | 1211(17.34) | 595(17.02) |  |
| 4 ≤ Day < 8 | 1630(29.18) | 419(29.99) |  | 2049(29.34) | 1160(33.19) |  |
| Day ≥ 8 | 2018(36.13) | 519(37.15) |  | 2537(36.33) | 1125(32.19) |  |
| **Level of surgery complexity, n (%)** |  |  | 0.675 |  |  | <0.001 |
| 1 | 169(3.03) | 49(3.51) |  | 2118(3.12) | 27(0.77) |  |
| 2 | 1143(20.46) | 288(20.62) |  | 1431(20.49) | 664(19.00) |  |
| 3 | 2333(41.77) | 593(42.45) |  | 2926(41.90) | 1311(37.51) |  |
| 4 | 1941(34.75) | 467(33.43) |  | 2418(34.48) | 1493(42.72) |  |
| **Type of prescription, n (%)** |  |  | 0.838 |  |  | <0.001 |
| Stat | 2296(41.10) | 570(40.80) |  | 2866(41.04) | 1145(32.76) |  |
| Long-term | 3290(58.90) | 827(59.20) |  | 2137(58.96) | 2350(67.24) |  |
| **Number of concurrent medicines, n (%)** |  |  | 0.745 |  |  | <0.001 |
| 1 | 868(15.54) | 203(14.53) |  | 2081(15.34) | 956(27.35) |  |
| 2 | 3932(70.39) | 1003(71.80) |  | 2935(70.67) | 1864(53.33) |  |
| 3 | 363(6.50) | 90(6.44) |  | 2533(6.49) | 354(10.13) |  |
| ≥ 3 | 423(7.57) | 101(7.23) |  | 2244(7.50) | 321(9.18) |  |
| **Type of medication, n (%)** |  |  | 0.374 |  |  | <0.001 |
| Sensory organs | 8(0.14) | 4(0.29) |  | 2282(0.17) | 5(0.14) |  |
| Respiratory system | 60(1.07) | 19(1.36) |  | 2969(1.13) | 129(3.69) |  |
| Musculoskeletal agents | 601(10.76) | 165(11.81) |  | 2666(10.97) | 135(3.86) |  |
| Anti-infective drugs | 1785(31.95) | 457(32.71) |  | 2212(32.11) | 806(23.06) |  |
| Antineoplastic and immunomodulating agents | 101(1.81) | 27(1.93) |  | 128(1.49) | 36(1.03) |  |
| Genitourinary system and sex hormones | 42(0.75) | 10(0.72) |  | 2242(0.74) | 84(2.40) |  |
| Nervous system | 415(7.43) | 105(7.52) |  | 2240(7.45) | 236(6.75) |  |
| Endocrine system | 87(1.56) | 22(1.57) |  | 2089(1.56) | 45(1.29) |  |
| Digestive system and metabolism | 1603(28.70) | 400(28.63) |  | 2013(28.68) | 1368(39.14) |  |
| Cardiovascular system | 281(5.03) | 47(3.36) |  | 2228(4.70) | 264(7.55) |  |
| Blood and hematopoietic system | 290(5.19) | 75(5.37) |  | 2625(5.23) | 207(5.92) |  |
| Others | 313(5.60) | 66(4.72) |  | 379(5.43) | 180(5.15) |  |
| **Route of administration, n (%)** |  |  | 0.112 |  |  | <0.001 |
| Oral | 549(9.83) | 109(7.80) |  | 658(9.42) | 457(13.08) |  |
| Iv/Im/Sc | 4546(81.38) | 1171(83.82) |  | 2747(81.87) | 2565(73.39) |  |
| Inhalers | 38(0.68) | 10(0.72) |  | 48(0.69) | 103(2.95) |  |
| Other | 453(8.11) | 107(7.66) |  | 560(8.02) | 370(10.59) |  |
| **Grade of prescription, n (%)** |  |  | 0.983 |  |  | <0.001 |
| 5 | 4721(84.51) | 1181(84.54) |  | 2942(84.52) | 3124(89.38) |  |
| 6 | 865(15.49) | 216(15.46) |  | 2081(15.48) | 371(10.62) |  |
| **Type of** **PEEs, n (%)** |  |  | 0.984 |  |  | <0.001 |
| Contra-indicated drug | 175(3.13) | 48(3.44) |  | 2213(3.19) | 129(3.69) |  |
| Drug interactions | 86(1.54) | 23(1.65) |  | 2089(1.56) | 25(0.72) |  |
| Inappropriate dilution | 1121(20.07) | 280(20.04) |  | 2411(20.06) | 765(21.89) |  |
| Incompatibilities | 134(2.40) | 29(2.08) |  | 163(2.33) | 192(5.49) |  |
| Incorrect infusion rate or order | 76(1.36) | 22(1.57) |  | 2878(1.40) | 107(3.06) |  |
| Non-compliance with hospital policies | 50(0.90) | 11(0.79) |  | 2151(0.87) | 16(0.46) |  |
| Wrong duration of treatment | 156(2.79) | 40(2.86) |  | 2916(2.81) | 32(0.92) |  |
| Wrong utilisation | 3788(67.81) | 944(67.57) |  | 2732(67.76) | 2229(63.78) |  |
| **Prescriber’s seniority, n (%)** |  |  | 0.807 |  |  | <0.001 |
| Junior | 1971(35.28) | 506(36.22) |  | 2477(35.47) | 1695(48.50) |  |
| Intermediate | 1561(27.94) | 384(27.49) |  | 1945(27.85) | 1003(28.70) |  |
| Senior | 2054(36.77) | 507(36.29) |  | 2561(36.67) | 797(22.80) |  |
| **Prescriber’s gender, n (%)** |  |  | 0.730 |  |  | <0.001 |
| Female | 1497(26.80) | 368(26.34) |  | 1865(26.71) | 1083(30.99) |  |
| Male | 4089(73.20) | 1029(73.66) |  | 5118(73.29) | 2412(69.01) |  |
| **Pharmacist training, n (%)** |  |  | 0.155 |  |  | <0.001 |
| Yes | 4792(85.79) | 1219(87.26) |  | 6011(86.08) | 3495(100.00) |  |
| No | 794(14.21) | 178(12.74) |  | 972(13.92) | 0(0.00) |  |

Note: ^a^The derivation cohort included training and internal validation cohorts; *p* < 0.05 is considered statistically significant; Iv/Im/Sc: intravenous, intramuscular, or subcutaneous injections; PEEs: potential prescription errors; Upper and lower GIT: Upper and lower gastrointestinal tract.

**Supplemental Table 4:** Performance of the ML models for whether the physician accepts the pharmacist's decision.

| Model | AUC | Accuracy | F1 Score | Sensitivity | Specificity | PPV | NPV | Kappa |
| --- | --- | --- | --- | --- | --- | --- | --- | --- |
| RF | 0.893 | 0.805 | 0.797 | 0.870 | 0.755 | 0.736 | 0.881 | 0.612 |
| LightGBM | 0.797 | 0.749 | 0.710 | 0.697 | 0.791 | 0.723 | 0.769 | 0.490 |
| DT | 0.780 | 0.771 | 0.750 | 0.780 | 0.764 | 0.721 | 0.816 | 0.539 |
| XGBoost | 0.777 | 0.734 | 0.686 | 0.661 | 0.792 | 0.714 | 0.749 | 0.457 |
| GBM | 0.773 | 0.726 | 0.659 | 0.603 | 0.822 | 0.727 | 0.725 | 0.433 |
| ET | 0.708 | 0.655 | 0.642 | 0.704 | 0.617 | 0.590 | 0.726 | 0.314 |
| ANN | 0.694 | 0.656 | 0.612 | 0.617 | 0.686 | 0.606 | 0.696 | 0.303 |
| AdaBoost | 0.682 | 0.643 | 0.641 | 0.726 | 0.577 | 0.574 | 0.729 | 0.295 |
| SVM | 0.679 | 0.650 | 0.643 | 0.718 | 0.596 | 0.583 | 0.730 | 0.307 |
| LR | 0.676 | 0.636 | 0.634 | 0.718 | 0.571 | 0.568 | 0.721 | 0.281 |
| NB | 0.644 | 0.628 | 0.643 | 0.764 | 0.521 | 0.556 | 0.738 | 0.274 |

Note**:** AUC: area under the receiver-operating-characteristic curve; NPV: negative predictive value; PPV: positive predictive value; AdaBoost: adaptive boosting; RF: random forest; LightGBM: light gradient boosting machine; ANN: artificial neutral network; DT: decision tree; SVM: support vector machine; XGboost: eXtreme gradient boosting; GBM: gradient boosting machine; ET: extra tree; NB: Naive Bayes; LR: logistic regression; ML: machine learning**.**

**Supplemental Table 5:** Performance of the RF model with varied numbers of features for whether the physician accepts the pharmacist's decision.

| Feature number | AUC | Sensitivity | Specificity | PPV | NPV | Accuracy | Kappa | F1 Score |
| --- | --- | --- | --- | --- | --- | --- | --- | --- |
| 15 | 0.893 | 0.801 | 0.812 | 0.770 | 0.839 | 0.807 | 0.611 | 0.785 |
| 14 | 0.895 | 0.896 | 0.720 | 0.715 | 0.898 | 0.797 | 0.600 | 0.795 |
| 13 | 0.892 | 0.857 | 0.754 | 0.732 | 0.870 | 0.799 | 0.599 | 0.789 |
| 12 | 0.886 | 0.836 | 0.773 | 0.742 | 0.857 | 0.800 | 0.600 | 0.786 |
| 11 | 0.884 | 0.829 | 0.770 | 0.739 | 0.852 | 0.796 | 0.591 | 0.781 |
| 10 | 0.873 | 0.783 | 0.784 | 0.740 | 0.822 | 0.784 | 0.564 | 0.761 |
| 9 | 0.868 | 0.858 | 0.702 | 0.693 | 0.863 | 0.771 | 0.547 | 0.767 |
| 8 | 0.856 | 0.684 | 0.862 | 0.795 | 0.777 | 0.784 | 0.554 | 0.736 |
| 7 | 0.826 | 0.749 | 0.754 | 0.704 | 0.793 | 0.752 | 0.499 | 0.726 |
| 6 | 0.802 | 0.634 | 0.826 | 0.741 | 0.742 | 0.742 | 0.467 | 0.683 |
| 5 | 0.763 | 0.726 | 0.682 | 0.642 | 0.761 | 0.702 | 0.403 | 0.681 |
| 4 | 0.692 | 0.650 | 0.676 | 0.611 | 0.711 | 0.664 | 0.323 | 0.630 |
| 3 | 0.678 | 0.700 | 0.632 | 0.599 | 0.729 | 0.662 | 0.327 | 0.646 |

Note: AUC: area under the receiver-operating-characteristic curve; NPV: negative predictive value; PPV: positive predictive value; RF model: random forest model.

**Supplemental Table 6:** Delong comparison of different features under the RF model with 95% confidence intervals.

| Feature number | AUC | SE^a^ | 95%CI^b^ |
| --- | --- | --- | --- |
| 15 | 0.893 | 0.008 | 0.876-0.909 |
| 12 | 0.886 | 0.009 | 0.868-0.902 |
| 9 | 0.868 | 0.009 | 0.849-0.885 |
| 6 | 0.802 | 0.012 | 0.780-0.822 |
| 3 | 0.678 | 0.014 | 0.653-0.702 |

Note: ^a^DeLong et al., 1988; ^b^Binomial exact; AUC: area under the receiver-operating-characteristic curve; SE: Standard Error; 95%CI: 95% Confidence Interval; RF model: random forest model.

**Supplemental Table 7:** Performance of the final RF model for whether the physician accepts the pharmacist's decision occurred.

| Timing of whether the physician accepts the pharmacist's decision occurrence | Number | Event | Mean AUC | Sensitivity | Specificity | PPV | NPV | Accuracy | F1 score |
| --- | --- | --- | --- | --- | --- | --- | --- | --- | --- |
| 2019 | 2734 | 1230 | 0.917 ± 0.021 | 0.793 | 0.854 | 0.819 | 0.835 | 0.827 | 0.805 |
| 2020 | 4249 | 1839 | 0.890 ± 0.012 | 0.732 | 0.849 | 0.788 | 0.806 | 0.798 | 0.759 |

Note: AUC: area under the receiver-operating-characteristic curve; NPV: negative predictive value; PPV: positive predictive value; RF model: random forest model.

**Supplemental Table 8:** Comparison of metrics for internal and external validation of the RF model.

| Group | AUC | Accuracy | Precision | Sensitivity | F1 Score | Kappa | PPV | NPV | Specificity |
| --- | --- | --- | --- | --- | --- | --- | --- | --- | --- |
| Internal Validation | 0.886 | 0.8 | 0.742 | 0.836 | 0.786 | 0.6 | 0.742 | 0.857 | 0.773 |
| External Validation | 0.786 | 0.87 | 0.863 | 0.463 | 0.603 | 0.533 | 0.863 | 0.871 | 0.98 |

Note: AUC: area under the receiver-operating-characteristic curve; NPV: negative predictive value; PPV: positive predictive value; RF model: random forest model.





**Supplemental Figure 1:** SHAP summary dot plots of all features for the top five best-performing machine learning models. (A) RF; (B) XGBoost; (C) LightGBM; (D) GBM; (E) DT. These plots illustrate the feature importance rankings in the internal validation cohort for each machine learning model. As the SHAP value of a feature increases, the probability of whether the physician accepts the pharmacist's decision also increases. Each dot represents the SHAP value for an individual feature for a single data point. Therefore, each data point has a dot for each feature in each row. The actual value of the feature for each data point is indicated by the color of the dot: red indicates higher actual values, while blue indicates lower actual values. Dots are vertically stacked to show density.


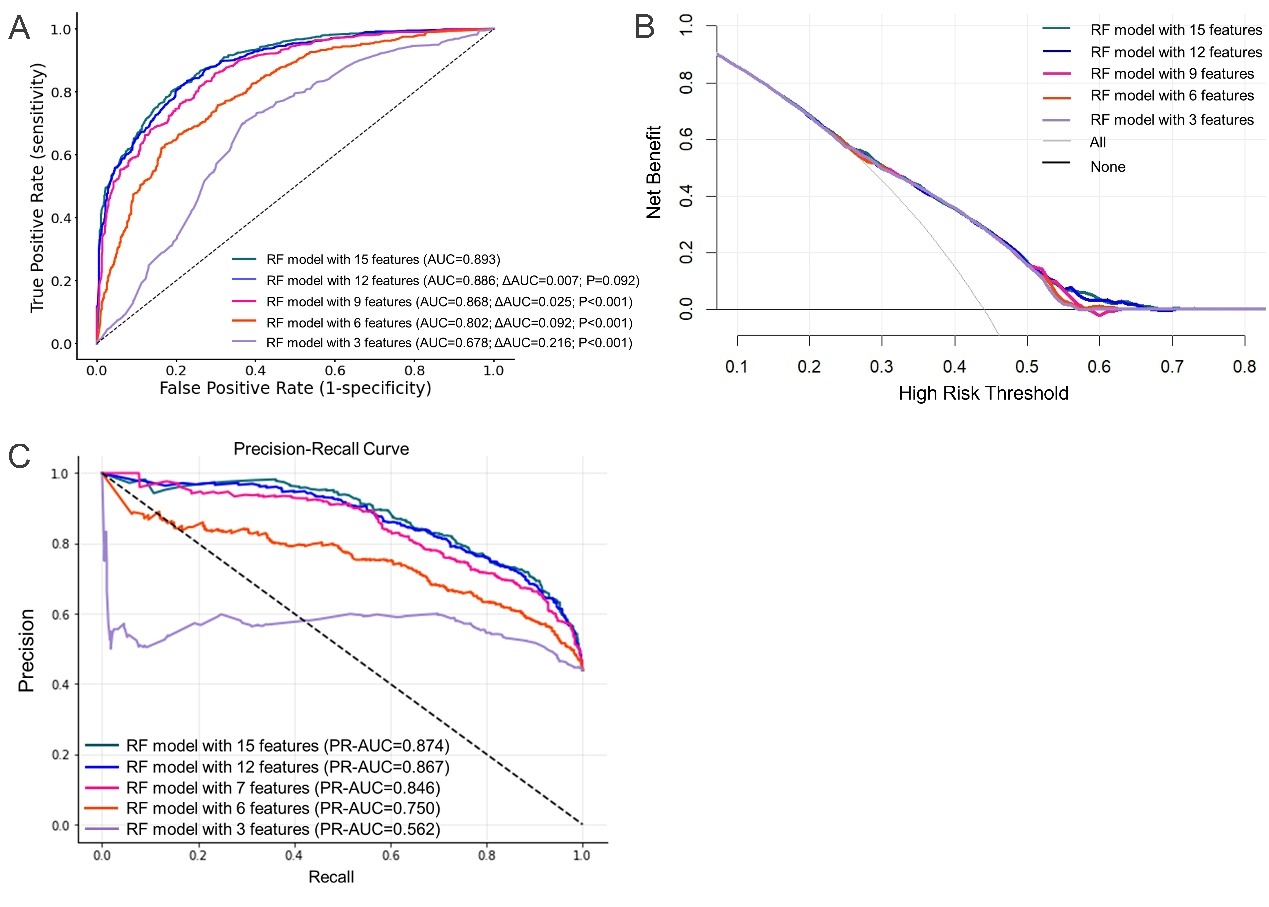


**Supplemental Figure 2**: Predictive Performance of the RF Model After Reducing Features. (A) ROC curves under different features. ΔAUC and P value represent the comparison with the RF model with 15 features. (B) DCA curves. (C) P-R curves. AUC: Area Under the ROC Curve; DCA: Decision Curve Analysis; P-R: Precision-Recall; RF: Random Forest; ROC: Receiver Operating Characteristic.





**Supplemental Figure 3:** Predictive performance of the final RF model in the cross validation. (A-B): Five-fold and ten-fold cross validation of the final RF model with 15 features. These plots represent the predictive performance in the cross-validation of the derivation cohort. AUC: area under the ROC curve; RF: random forest; ROC: receiver-operating-characteristic; SD: standard deviation.


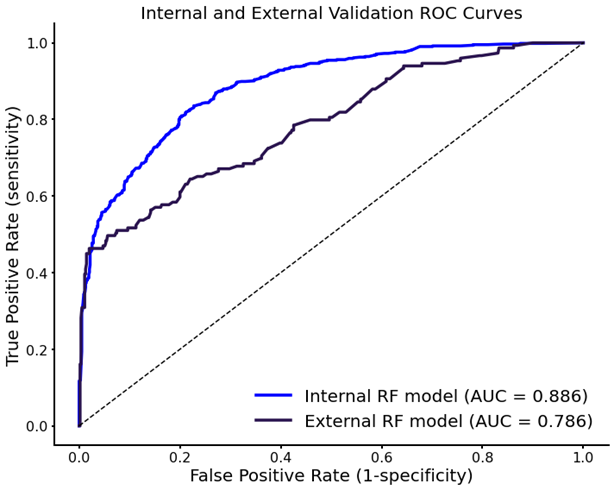


**Supplemental Figure 4**: Comparison of Internal and External Validation ROC Curves. RF: Random Forest; ROC: Receiver Operating Characteristic; AUC: Area Under the ROC Curve.


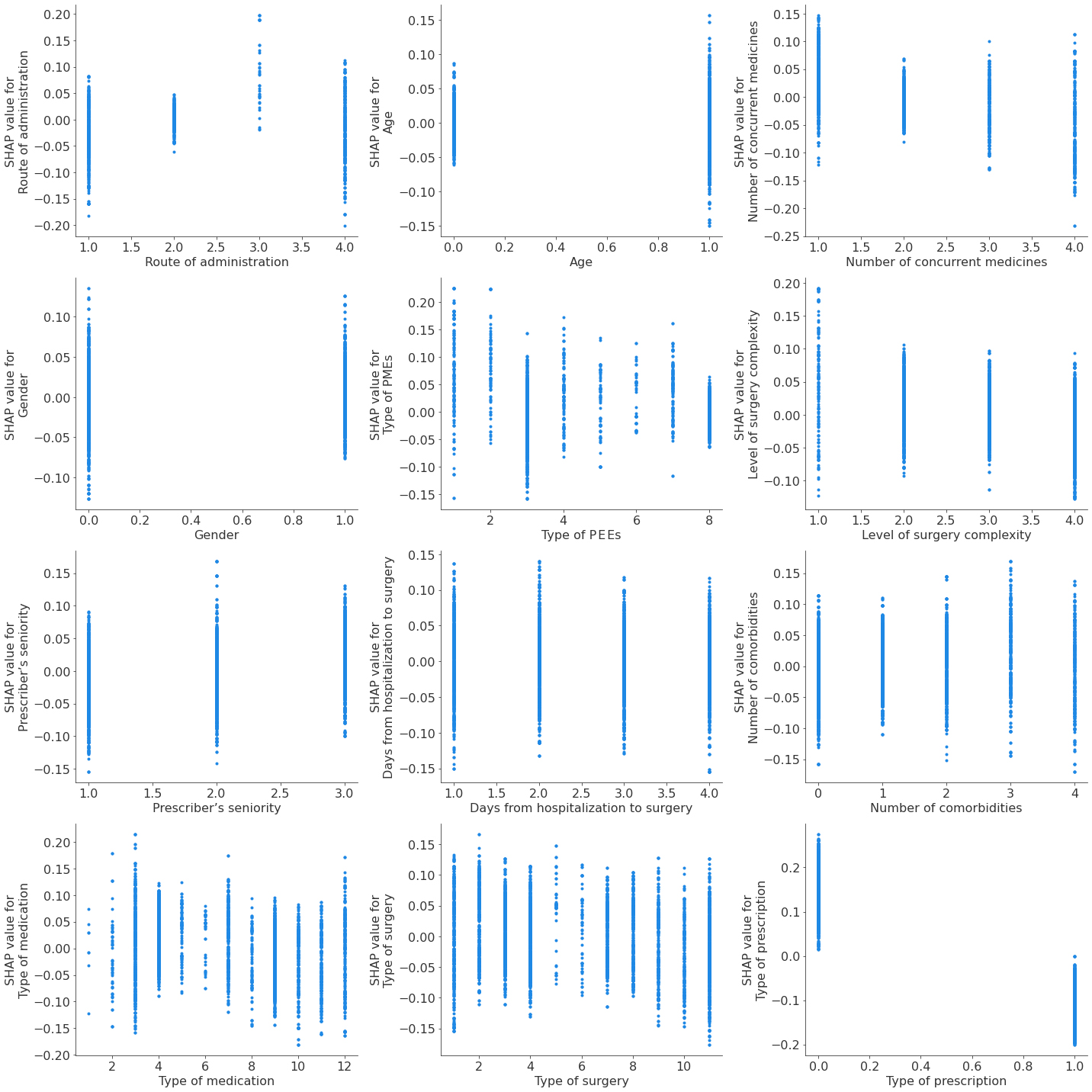


**Supplemental Figure 5:** SHAP Dependency Plots: Each dependency plot shows how a single feature affects the output of the predictive model. Each dot represents a single data point.
